# Characterization of the transporterB^0^AT3 (*Slc6a17*) in the rodent central nervous system

**DOI:** 10.1186/1471-2202-14-54

**Published:** 2013-05-14

**Authors:** Maria GA Hägglund, Sofie V Hellsten, Sonchita Bagchi, Anna Ljungdahl, Victor CO Nilsson, Sonja Winnergren, Olga Stephansson, Juris Rumaks, Simons Svirskis, Vija Klusa, Helgi B Schiöth, Robert Fredriksson

**Affiliations:** 1 Department of Neuroscience, Functional Pharmacology, Uppsala University, Uppsala, Sweden; 2 Department of Pharmacology, Faculty of Medicine, University of Latvia, Riga, Latvia

## Abstract

**Background:**

The vesicular B^0^AT3 transporter (SLC6A17), one of the members of the SLC6 family, is a transporter for neutral amino acids and is exclusively expressed in brain. Here we provide a comprehensive expression profile of B^0^AT3 in mouse brain using *in situ* hybridization and immunohistochemistry.

**Results:**

We confirmed previous expression data from rat brain and used a novel custom made antibody to obtain detailed co-labelling with several cell type specific markers. B^0^AT3 was highly expressed in both inhibitory and excitatory neurons. The B^0^AT3 expression was highly overlapping with those of vesicular glutamate transporter 2 (VGLUT2) and vesicular glutamate transporter 1 (VGLUT1). We also show here that *Slc6a17*mRNA is up-regulated in animals subjected to short term food deprivation as well as animals treated with the serotonin reuptake inhibitor fluoxetine and the dopamine/noradrenaline reuptake inhibitor bupropion.

**Conclusions:**

This suggests that the B^0^AT3 transporter have a role in regulation of monoaminergic as well as glutamatergic synapses.

## Background

The SLC6 family of proteins consists of 19 functional members and four pseudogenes in human [1] and many of the proteins belonging to the this family are presynaptic reuptake transporters. The major substrates are the monoamine neurotransmitters noradrenaline (NA), dopamine (DA) and serotonin (5-HT), amino acids including γ-aminobutyric acid (GABA) as well as other neutral amino acids, and osmolytes such as taurine, creatine and betaine [2,3]. Transporters from the SLC6 family are secondary active transporters dependent on electrochemical Na^+^ or H^+^gradients to drive the transport. Na^+^ gradients are maintained over the plasma membrane of all cells, while H^+^ gradients are maintained over internal membranes such as synaptic vesicles. Already in the beginning of the 1990s, the sequence of the SLC6A17 transporter was determined by sequencing cDNA from mammalian sources and was shown to have high expression exclusively in the CNS. At that time, the substrate of transport was not known [4-6]. Phylogenetic analysis of the whole SLC6 family originally classified the members into four subgroups termed GABA, monoamine, amino acid, and the orphans [1,7]. The orphan subgroup is now known to contain transporters for neutral amino acids and is hence renamed the amino acid transporter group II [2]. SLC6A17 is most similar to SLC6A15 (B^0^AT2) regarding sequence identities [1,2,8], mRNA expression [9], as well as substrate profile [8,10-12]. The protein product of the SLC6A17 gene is termed B^0^AT3 [12], which will be the notation used further in this paper. Interestingly, the main difference between B^0^AT2 and B^0^AT3 is the subcellular localization, with B^0^AT2 being localized at the plasma membrane (unpublished data), while B^0^AT3 is known to be expressed on vesicles [11-14].

It was recently shown by two independent groups that B^0^AT3 acts as a transporter of a broad range of neutral amino acids with affinity for proline, leucine and glycine, alanine and glutamine [11,12]. However, the preference for ion coupling of B^0^AT3 is arguable as one study showed that B^0^AT3 is Na^+^-dependent and Cl^-^-independent [12], while another study showed B^0^AT3 not to be Na^+^dependent but rather dependent on the H^+^gradient maintained by the vacuolar-type H^+^-ATPase [11].

*In situ* hybridization and immunohistochemistry studies, performed mainly on rat tissues, have revealed that *Slc6a17* mRNA as well as the B^0^AT3 protein is widely distributed throughout the CNS. The transporter is found exclusively in axon terminals of most glutamatergic neurons and in a sub-population of GABAergic neurons in embryonic [15] as well as adult rat brain [4,9,13,14,16-18]. A similar pattern have been suggested also in mouse [19] and human [20], although no comprehensive mapping have been performed in these species. The physiological function of B^0^AT3 (SLC6A17) is still unknown, although several alternatives have been suggested [11,12,14,19]. Many of the amino acid transporters in the SLC6 family are known to play important roles in several pathological conditions including obesity (SLC6A14) [21-23] and major depression (SLC6A15) [24]. Providing that B^0^AT3 has a very similar substrate profile as B^0^AT2, but with unique expression in the synapses, we hypothesized that B^0^AT3 could also play a role in depression and in the action of antidepressant drugs. Given the proposed synaptic localization, B^0^AT3 could possibly play a role in synaptic remodeling, a process important in the long term action of antidepressant drugs [25] as well as in other functions of the nervous system.

In this context, we challenged the serotonin and the dopamine/noradrenaline systems with drugs (fluoxetine and bupropione, respectively) and studied effects on expression of *Slc6a17* and *Slc6a15* mRNA in various brain regions. Fluoxetine is an antidepressant drug of the selective serotonin reuptake inhibitor (SSRI) class, clinically used to treat depressive disorders, while bupropion is a noradrenaline and dopamine reuptake inhibitor. Bupropion is used in treatment of depression as well as a smoking cessation aid, due to its actions on the reward system in the brain. We also studied *Slc6a17* and *Slc6a15* transporters in terms of their involvement in food intake control in a model of acute food deprivation and in a model for chronic food restriction, using a validated quantitative real-time PCR method. We show here that *Slc6a17*mRNA is strongly regulated under these conditions. Additionally, we provide a complete view of B^0^AT3 (*Slc6a17*) expression in the central nervous system of mouse. We accomplished this with *in situ* hybridization on mouse brain and spinal cord, confirming previously shown gene expression of *Slc6a17*. We also made polyclonal B^0^AT3 antibody and used immunohistochemistry to study endogenous protein expression of B^0^AT3 in mouse nervous system, to identify specific cell types expressing the B^0^AT3 transporter.

## Results

### High Slc6a17 gene expression in rat CNS

**Figure 1:**
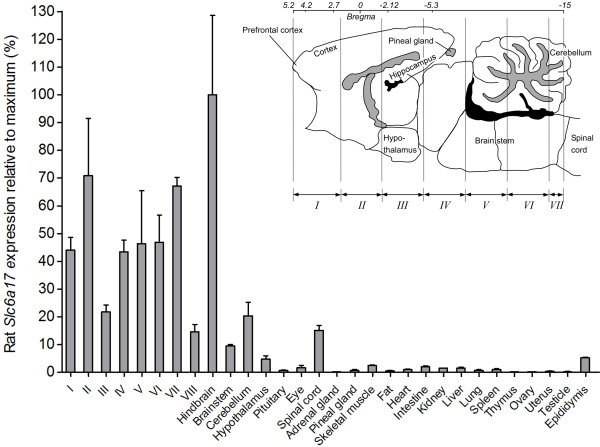
**High mRNA expression of *Slc6a17 *in rat CNS and low expression in periphery.** Real-time PCR data visualized as column charts with standard deviations displaying the rat *Slc6a17* expression (% ± SD%) relative to maximum (fold decrease). *Slc6a17* showed high cDNA expression in brain, spinal cord and epididymis, and low or almost no expression in the other peripheral tissues. The abbreviations I–VIII indicates eight rat brain cross sections and the picture with the sagittal mouse brain indicates the Bregma coordinates for these sections.

Expression analysis of *Slc6a17* in CNS and peripheral tissues (Figure 1) showed widespread, multifocal expression in the rat CNS and low or almost no expression in peripheral tissues. The relative expression of *Slc6a17* was highest in hindbrain (100 ± 29), brain slice II (71 ± 21) and brain slice VII (67 ± 3). *Slc6a17*mRNA was also highly expressed in the other tissues with the following relative expression; brain slice VI (47 ± 10), brain slice V (46 ± 19), brain slice I (44 ± 5), brain slice IV (43 ± 4), brain slice III (22 ± 2), cerebellum (20 ± 5), spinal cord (15 ± 2), brain slice VIII (15 ± 3), brain stem (9 ± 1), epididymis (5 ± 0) and hypothalamus (5 ± 1). Relative expression below 2% was seen in skeletal muscle, intestine, liver and eye, while really low expression below 1% was seen in the other peripheral tissues (Figure 1).

### Expression of Slc6a17 mRNA in mouse POMC and NPY neurons, and in both excitatory and inhibitory neurons

**Figure 2:**
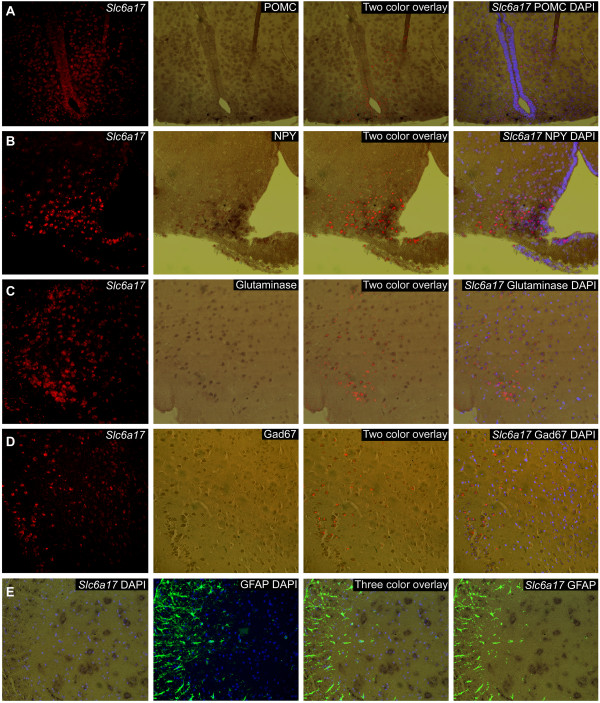
**Expression of *Slc6a17*mRNA in POMC, NPY, excitatory and inhibitory neurons.** Double *in situ* hybridization **(A-D)** and combined *in situ* hybridization/immunohistochemistry **(E)** using *Slc6a17* probe for co-localization of *Slc6a17*mRNA and markers in mouse brain and spinal cord are shown in this figure. The cell nucleus marker DAPI was stained in blue. **(A)** Co-localization of *Slc6a17*mRNA (red) and POMC-mRNA (dark blue) in hypothalamus close to the third ventricle (Bregma −2.18). **(B)** Overlapping expression of *Slc6a17* (red) mRNA and NPY labeled neurons (dark blue) in arcuate nucleus in hypothalamus (Bregma −2.06). **(C)** Co-localization of *Slc6a17*mRNA (red) and glutaminase-mRNA (brown) in excitatory neurons in cerebral cortex (Bregma −1.58). **(D)** Co-expression of *Slc6a17*mRNA (red) and Gad67-mRNA (brown) in inhibitory neurons in cerebral cortex (Bregma −1.58). **(E)** No co-localization between *Slc6a17*mRNA (brown) and the GFAP protein (green) expression in spinal cord (L2). All pictures were taken with 20x objective using cryo sections (**A** and **B**) and paraffin embedded sections (**C**-**E**). Bregma coordinates are according to Franklin and Paxinos 2007 [28].

Double *in situ* hybridization was used to identify cell types expressing *Slc6a17* in mouse brain (Figure 2A-D). Proopiomelanocortin (POMC) and neuropeptide Y (NPY) are expressed in adjacent subpopulations of arcuate nucleus neurons (Arc), and are known to be involved in the regulation of food intake [26]. Our experiments demonstrated that *Slc6a17* mRNA co-localized with POMC and other neurons in Arc in the hypothalamus (Figure 2A). The *Slc6a17* mRNA also co-localized with NPY and was also found in other neurons in Arc (Figure 2B). *Slc6a17* showed overlapping mRNA expression with glutaminase, but was also found in glutaminase negative neurons in cerebral cortex (Figure 2C). *Slc6a17* also localized to Gad67 expressing neurons as well as other neurons in cortex (Figure 2D). These results collectively show that *Slc6a17* is expressed in both excitatory and inhibitory neurons in the brain. Combined *in situ* hybridization with immunohistochemistry was used on mouse spinal cord sections to investigate if *Slc6a17* was expressed in GFAP positive (glia) cells [27] (Figure 2E). The *Slc6a17* mRNA expression was located to interneurons in the grey matter and in subsets of cholinergic motor neurons in the upper lumbar vertebrae L2. Co-staining with *Slc6a17* mRNA and the GFAP antibody did not overlap in spinal cord, suggesting no *Slc6a17* expression in astrocytes.

### Abundant mRNA expression of Slc6a17 in mouse brain and spinal cord

**Figure 3:**
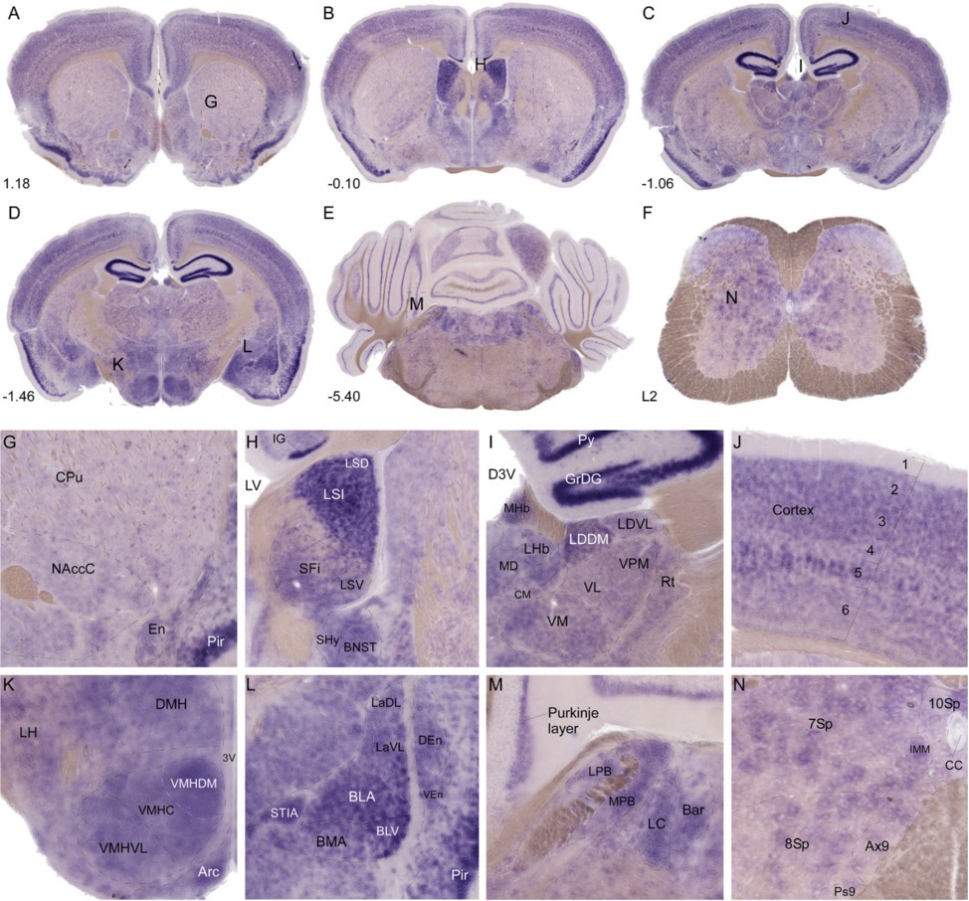
***Slc6a17*mRNA expression in mouse CNS.** In situ hybridization on free floating sections using 1 μg probe/ml of DIG-labeled (blue staining) mouse Slc6a17 probe. Coronal mouse brain and spinal cord sections, visualized as over view (A-F) and close up (G-N) pictures. Brain abbreviations; caudate putamen (striatum) (CPu), nucleus accumbens, core (NAccC), lateral septal nucleus, lateral ventricle (LV), indusium griseum (IG), dorsal part (LSD), lateral septal nucleus, intermediate part (LSI), lateral septal nucleus, ventral part (LSV), septofimbrial nucleus (SFi), bed nucleus of the striater minalis (BNST), septohypothalamic nucleus (SHy), pyramidal cell layer of the hippocampus (Py), granule cell layer of the dentate gyrus (GrDG), medial habenular nucleus (MHb), lateral habenular nucleus (LHb), mediodorsal thalamic nucleus (MD), laterodorsal thalamic nucleus, dorsomedial part (LDDM), laterodorsal thalamic nucleus, ventrolateral part (LDVL), central medial thalamic nucleus (CM), ventromedial thalamic nucleus (VM), ventrolateral thalamic nucleus (VL), ventral posteromedial thalamic nucleus (VPM), reticular thalamic nucleus (Rt), layers of cortex (1–6), third ventricle (3 V), dorsomedial hypothalamic nucleus (DMH), ventromedial hypothalamic nucleus, dorsomedial part (VMHDM), ventromedial hypothalamic nucleus, central part (VMHC), ventromedial hypothalamic nucleus, ventrolateral part (VMHVL), lateral hypothalamus (LH), arcuate hypothalamic nucleus (Arc), lateral amygdaloid nucleus, dorsolateral part (LaDL), lateral amygdaloid nucleus, ventrolateral part (LaVL), basolateral amygdaloid nucleus, anterior part (BLA), basolateral amygdaloid nucleus, ventral part (BLV), bed nucleus of the striater minalis, intra amygdaloid (STIA), basomedial amygdaloid nucleus, anterior part (BMA), dorsal endopiriform nucleus (DEn), ventral endopiriform claustrum (VEn), piriform cortex (Pir), lateral parabrachial nucleus (LPB), medial parabrachial nucleus (MPB), locus coeruleus (LC), barrington’s nucleus (Bar). Abbreviations and described spinal cord regions; vertebrae lumbales 2 (L2), central canal (CC), intermediomedial column (IMM), lamina 7 of the spinal gray (7Sp), lamina 8 of the spinal gray (8Sp), lamina 10 of the spinal gray (10Sp), axial muscle motoneurons of lamina 9 (Ax9), psoas motoneurons of lamina 9 (Ps9). Bregma levels, and described brain and spinal cord regions according to Franklin and Paxinos 2007 [28] and Allen Mouse Brain Atlas [29].

*In situ* hybridization with a probe against *Slc6a17* was used to evaluate expression of *Slc6a17* in mouse brain and spinal cord (Figure 3, Additional file 1: Table S1). The DIG-labeled antisense *Slc6a17* probe was 285 bp long and directed against the last coding exon of the gene. We confirmed high levels of *Slc6a17* expression in gray but not in the white matter in the brain. High mRNA expression was seen in hippocampus, cerebellum, cerebral cortex, thalamus, amygdala, pons, while low expression was seen in basal ganglia and spinal cord. *Slc6a17* showed expression in the strias of CPu (Figure 3A and G) and in NAcc. Staining was detected in septum (Figure 3B and H), hippocampal GrDG and Py (Figure 3C and I), all layers of cerebral cortex except layer 1 (Figure 3C and J), DMH, VMH, LH and Arc in hypothalamus (Figure 3D and K), BLA and BLV in amygdala (Figure 3D and L), LC and Bar in pons, and in the Purkinje layer of cells in cerebellum (Figure 3E and M). In addition, *Slc6a17* was also highly expressed in Piriform cortex. Widespread *Slc6a17* mRNA expression was detected in the spinal cord, where the gene was detected in subsets of somatic motor neurons and in interneurons (Figure 3F and N).

### Specificity of the B^0^AT3 antibody

We generated rabbit polyclonal B^0^AT3 antibody against an epitope in the N-terminus, to verify the expression obtained with the commercially available antibody. We used immunohistochemistry to document the epitope specificity of the antibody by co-labeling it with the commercially available monoclonal mouse-B^0^AT3 antibody (Additional file 1: Figure S1). The immunohistochemistry with the B^0^AT3 antibodies showed highly overlapping expression in mouse CNS, with one exception. The commercially available B^0^AT3 antibody gave staining within some cells in spinal cord which was not seen for the custom-made polyclonal B^0^AT3 antibody. This could be due to the fact that different epitopes were used to generate the antibodies and that, the epitope we used is not exposed in these specific cells. It could also be due to low expression of B^0^AT3 in these cells and that the mouse antibody has higher sensitivity. Another possibility is that the commercial antibody is slightly less specific and hence detects another protein in these cells. This experiment verifies the specificity of both antibodies.

### Mouse CNS localization of B^0^AT3 protein in the cell body, axon and synapses of inhibitory and excitatory neurons

**Figure 4:**
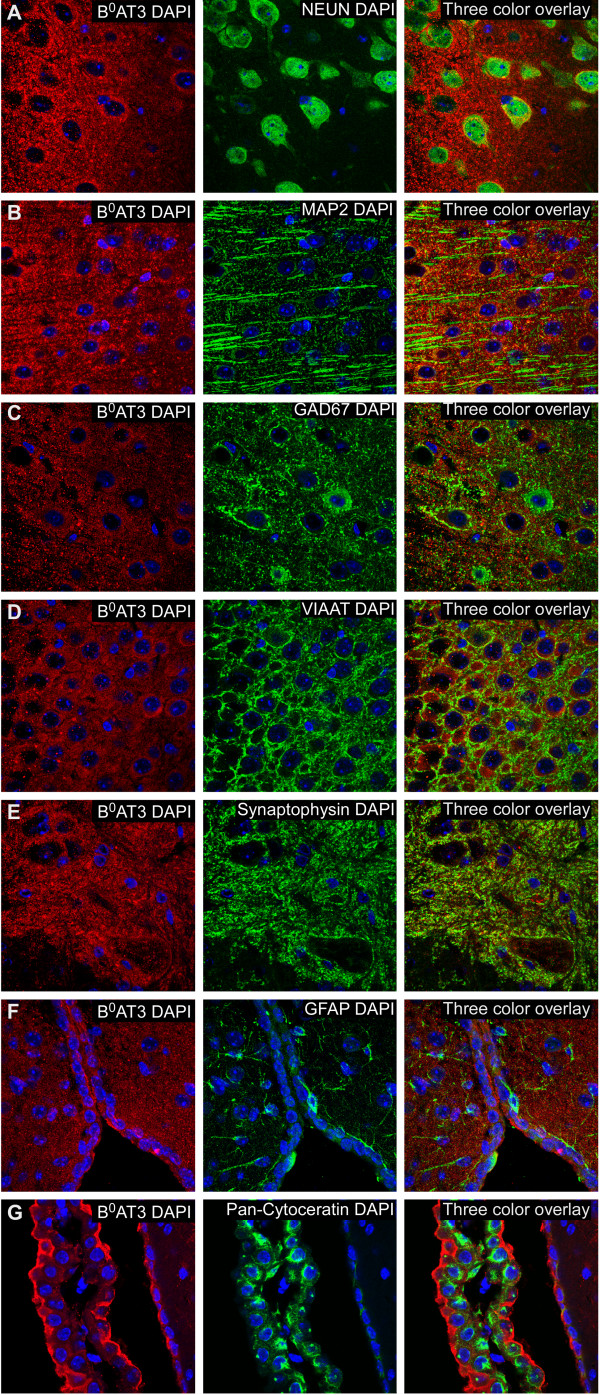
**B**^**0**^**AT3 immunoreactivity in vesicles, GABAergic and other neurons.** Immunohistochemistry on mouse brain and spinal cord with B^0^AT3 immunoreactivity (red), markers (green), and nucleus marker DAPI (blue). **(A)** B^0^AT3 was extensively expressed in cortex (Bregma 0.02) together with NeuN positive cells and found in the soma and axon **(B)** The neuronal marker MAP2 and B^0^AT3 were found together in cortex (Bregma −0.70). **(C)** The immunoreactivity of the GABAergic marker Gad67 overlapped with the expression of the B^0^AT3 transporter in cortex (Bregma −0.10). **(D)** Highly overlapping expression was seen for B^0^AT3 and the presynaptic GABAergic neuronal marker VIAAT in hippocampus (Bregma −3.40). **(E)** Immunoreactivity of B^0^AT3 and the vesicular marker synaptophysin extensively overlapped in spinal cord (upper vertebrae L2 lumbar). **(F)** No overlap was seen with the astrocyte marker GFAP and B^0^AT3 in hypothalamus (Bregma −0.82). **(G)** No protein overlap was seen between B^0^AT3 and pan-cytokeratin (Bregma −2.30, around the third ventricle). Confocal pictures on paraffin sections taken with 63x magnification.

**Figure 5:**
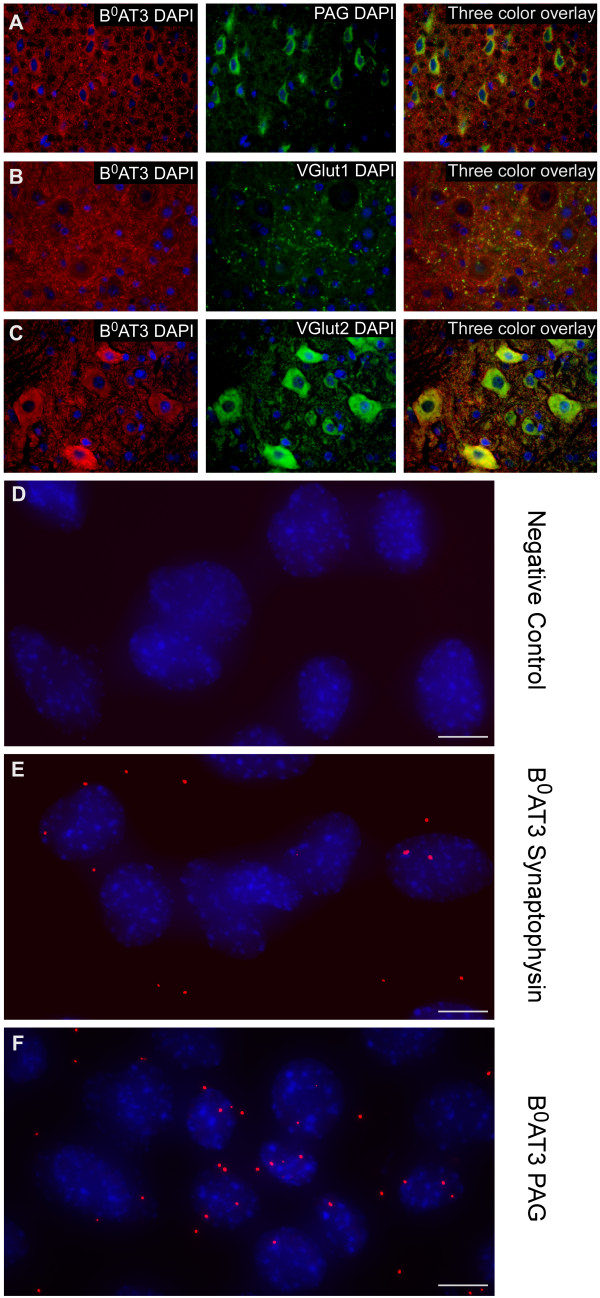
**Protein localization and interactions of B**^**0**^**AT3 to glutamatergic neurons and vesicles.** Immunohistochemistry **(A-C)** on mouse brain and spinal cord and proximity ligation assay (PLA) **(D-E)** on a hypothalamic mouse cell line are illustrated here with B^0^AT3 immunoreactivity in red, markers in green, and nucleus marker DAPI in blue. **(A)** Highly overlapping expression in glutamatergic neurons between B^0^AT3 and the enzyme PAG in cerebral cortex in the brain (Bregma 1.18). **(B)** Low overlapping expression for the vesicular marker VGLUT1 and B^0^AT3 in dorsal horn in spinal cord (upper vertebrae L2 lumbar). **(C)** Expression of the vesicular glutamate transporter VGLUT2 extensively overlapped with the expression of the B^0^AT3 transporter in ventral horn in spinal cord (Bregma −0.10). Fluorescent micrographs of paraffin sections taken with 40x magnification. PLA signals (red fluorescent amplification product) and the nuclei (blue) are shown as an increased intensity projection of the raw image based on 20 z-planes **(D-F)**. **(D)** No interactions were seen in the negative control that was run without primary antibodies. **(E)** Moderate number of interactions detected between B^0^AT3 and synaptophysin. **(F)** High number of interactions detected between B^0^AT3 and PAG (scale bar, 10 μm).

A number of neuronal markers were used to visualize the neuronal expression of the B^0^AT3 transporter (Figures 4 and 5). Highly overlapping expression was seen for B^0^AT3 and the neuronal marker (NeuN) [30] (Figure 4A). The B^0^AT3 expression was localized to the cell body and in the axon of neurons in cerebral cortex and also in other areas. The microtubule-associated protein 2 (MAP2) [31] and B^0^AT3 co-localized in the cell body of neurons in the cerebral cortex and other areas in the brain (Figure 4B). Gad67 [32] was used to show expression of B^0^AT3 in GABAergic neurons (Figure 4C). We also used the vesicular inhibitory amino acid transporter VIAAT (VGAT), a marker of synaptic vesicles in GABAergic neurons and in sets of glycinergic neurons [33], and synaptophysin, a generally expressed presynaptic vesicle glycoprotein [34], to investigate the vesicular localization of B^0^AT3. Co-localization of B^0^AT3 and VIAAT indicated highly overlapping expression in inhibitory synapses in hippocampus, and similar co-localization was also found in other areas of the brain and in spinal cord (Figure 4D). B^0^AT3 and synaptophysin immunoreactivity extensively overlapped in grey matter in spinal cord, and similar expression pattern was also seen in brain (Figure 4E). As expected, the B^0^AT3 transporter did not co-localize with the astrocyte marker GFAP [27] in hypothalamus or in other areas of the brain (Figure 4F). Interestingly, the choroid plexus epithelial cell marker pan-cytokeratin [35] localized in cells surrounding the ventricles, towards the cerebrospinal fluid (CSF), while B^0^AT3 was found in the same cells but on the opposite side (Figure 4G). The protein expression of B^0^AT3 and pan-cytokeratin did not overlap in the lateral ventricle (LV) or in any of the other ventricles in brain. Additionally, markers for phosphate-activated glutaminase (PAG), the enzyme which generates glutamate and ammonia from glutamine [36,37], and vesicular glutamate transporters (VGLUT1 and VGLUT2) [38-40] were used to investigate the expression of B^0^AT3 in excitatory neurons. Highly overlapping expression was seen for B^0^AT3 and PAG in cortex and in cells close to the 3 V in hypothalamus in the brain (Figure 5A). Moderate overlapping expression was seen for VGLUT1 and B^0^AT3 in the gray matter of dorsal horn in spinal cord and in the brain (Figure 5B), while extensively overlapping expression was seen for B^0^AT3 and VGLUT2 in gray matter of ventral horn in spinal cord and in the cortex in the brain (Figure 5C). The VGLUT2 staining shown here indicates synaptic staining as well as staining on the cell-bodies, which should be interpreted with caution as this is normally not seen and could be the result of non-specific staining. Similar overlap was also seen between VGLUT1 (Additional file 1: Figure S2A) as well as between VGLUT2 and B^0^AT3 (Additional file 1: Figure S2B) in the brain. To conclude, B^0^AT3 is located on GABAergic and glutamatergic neurons with expression in synapses in the brain and in spinal cord.

### Protein interactions between B^0^AT3 and synaptic vesicles and between B^0^AT3 and glutaminase

The proximity ligation assay (PLA) technology was used for detection of interactions between antibodies targeting B^0^AT3 and the vesicle marker (synaptophysin), and between B^0^AT3 and the glutaminase marker PAG in hypothalamic mouse cells (Figure 5). No protein interactions were seen in the negative control (Figure 5D). A moderate number of interactions were seen between ^0^B^0^AT3 and synaptophysin (Figure 5E), while a higher number of interactions was seen between B^0^AT3 and PAG.

### Cellular localization of B^0^AT3 immunoreactivity in neurons and synapses in primary cell culture

**Figure 6:**
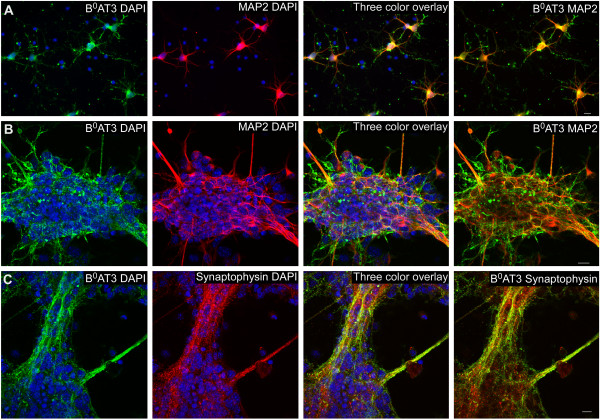
**B**^**0**^**AT3 immunoreactivity co-localized with neuronal and vesicle markers in primary cell cultures.** Immunohistochemistry was performed on embryonic mouse E13 primary cell cultures with B^0^AT3 antibody (green), markers (red) and nucleus marker DAPI (blue). **(A)** Highly overlapping expression of B^0^AT3 and the neuronal marker MAP2 in the cell body close to the membrane and on dendrites in single cells from whole brain. Large nucleus belong to neuronal cells labelled with MAP2, while small nucleus represent glial cells. **(B)** Overlapping expression of B^0^AT3 and MAP2 in neuronal axons on cells from fore brain. **(C)** Co-localization of B^0^AT3 and the vesicular marker synapophysin on cells from fore brain (scale bar, 10 μm).

Immunohistochemistry was used to investigate neuronal and vesicular localization of B^0^AT3 in mouse E13 primary cell cultures (Figure 6). The immunoreactivity of B^0^AT3 and the neuronal marker microtubule-associated protein 2 (MAP2) [31] highly overlapped in the cell body close to the membrane and in the dendrites of single cells from whole brain (Figure 6A). Only cells containing large nuclei were labelled with MAP2, while cells with smaller nuclei, representing non-neuronal cells, were not labelled with either the neuronal marker or B^0^AT3. In the next experiment, cells were grown for longer time prior to immunohistochemistry to be able to study the B^0^AT3 expression in colonies of more matured cells. B^0^AT3 immunofluorescence indicated expression in the neuronal axons on cells from fore brain (Figure 6B). To further visualize the vesicular localization of B^0^AT3 the vesicular marker synaptophysin [34] was co-labelled with B^0^AT3 on cells from fore brain. The B^0^AT3 and synaptophysin proteins were highly overlapping in the cell bodies and in the axons.

### Up-regulation of Slc6a17 in hypothalamus in food restricted rats

**Figure 7:**
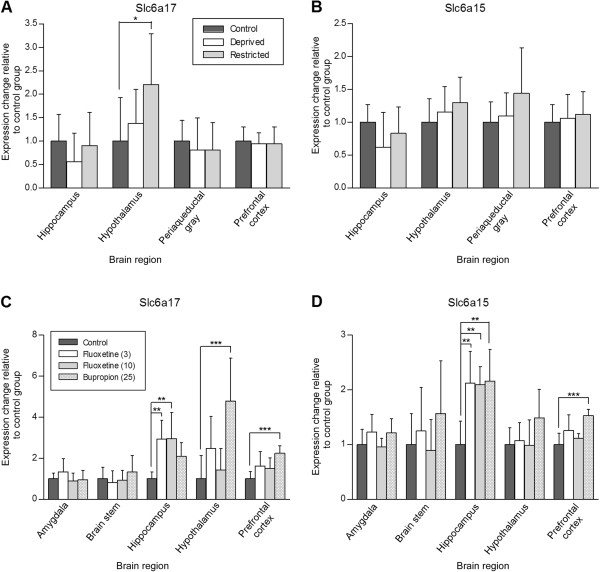
**Changes in *Slc6a17 *expression levels in hypothalamus in overnight starved and long term food restricted (45% of *ad lib *food intake), as well as in hippocampus and hypothalamus in a model for the monoamine system. (A-B)** Three groups (n = 8 rats/group) were compared within a control group with free access to food (dark grey), a food deprived group (white), and a food restricted group (light grey). **(A)** In the restricted animals, the gene expression of *Slc6a17* significantly (p = 0.0461) was up-regulated in hypothalamus. **(B)** The *Slc6a15* gene was not significantly up- or down-regulated in any of the studied tissues. **(C-D)** Four groups (n = 8 rats/group) were compared within a control group given saline (dark gray), a group given 3 mg/kg fluoxetine (white), a group given 10 mg/kg fluoxetine (light grey) and a group given 25 mg/kg bupropion (black dots). **(C)** The *Slc6a17* gene was significantly (p = 0.0009) up-regulated in hippocampus after low and high dose of fluoxetine application, while a non significant up-regulation was seen after bupropion treatment. Bupropion injection caused a significant up-regulation of *Slc6a17* in hypothalamus (p = 0.0002) and in prefrontal cortex (p = 0.0005). **(D)** Significant (p = 0.0006) up-regulation of *Slc6a15* was seen in hippocampus after injection of both doses of fluoxetine and bupropion and the *Slc6a15* gene was significantly (p = 0.0002) up-regulated in prefrontal cortex after bupropion treatment. All data were normalized to the mean value for the control group. Significance levels were obtained from one-way ANOVA followed by Tukey’s post hoc test on a data set with p < 0.05 from the ANOVA. Multiple comparison test significant levels are indicated by asterisks (*, p < 0.05; **, p < 0.01; ***, p < 0.001).

Changes in expression levels of *Slc6a17* and *Slc6a15* were investigated in a model for appetite where rats were acutely food deprived for 48 hours (short term starvation) or 45% chronic food restricted during 12 days (long term starvation) and compared with an *ad libitum* fed control group (Figure 7A-B). Expression levels were studied in a number of tissues; hippocampus, hypothalamus, periaqueductal gray and prefrontal cortex. A significant (p = 0.0461) up-regulation was seen for *Slc6a17* in hypothalamus after short term starvation, and a trend of up-regulation was seen after long term starvation (Figure 7A). This suggests that *Slc6a17 has a more pronounced role in anticipation of food than in appetite regulation.* No significant differences in expression were seen for *Slc6a15* in any of the studied tissues (Figure 7B), and no significant up-regulation was seen for the *Slc6a15* gene in food restricted rats.

### Up-regulation of Slc6a17 and Slc6a15 in rats treated with antidepressant drugs

The monoamine system was challenged with antidepressants to study the serotonin (fluoxetine) and the dopamine/noradrenaline systems (bupropion) (Figure 7C-D). Changes in mRNA expression of *Slc6a17* and *Slc6a15* transporters as response to the drugs were investigated in a number of tissues (amygdala, brain stem, hippocampus, hypothalamus and prefrontal cortex). *Slc6a17* was significantly up-regulated in hippocampus independent of the dose of fluoxetine (p = 0.0009), while a trend of up-regulation was seen after bupropion treatment (Figure 7C). Bupropion injection caused a significant up-regulation of *Slc6a17* in hypothalamus (p = 0.0002) and in prefrontal cortex (p = 0.0005). No changes in gene expression were seen in amygdala and brainstem. Interestingly, a similar pattern was seen for the related *Slc6a15* transporter, with up-regulation in hippocampus after injection of both doses of fluoxetine and the bupropion antidepressant (p = 0.0006) (Figure 7D). The *Slc6a15* gene was also significantly up-regulated in prefrontal cortex after bupropion treatment (p = 0.0002).

## Discussion

In this study we used quantitative RT-PCR (qPCR) in a large panel of adult rat brain and peripheral tissues (Figure 1), to further refine the expression profile of the *Slc6a17* gene. The highest expression levels of *Slc6a17* mRNA were found in hindbrain, various brain cross sections, cerebellum, spinal cord, brain stem and hypothalamus, while very low or no expression was seen in the peripheral tissues with the exception of epididymis. Consequently, the *Slc6a17* transporter is highly and selectively expressed in the CNS of adult rat.

Abundant mRNA expression of *Slc6a17* in adult and embryonic rat CNS has previously been shown using *in situ* hybridization. Consistent results indicated restricted expression exclusively in neurons, both glutamatergic and subsets of GABAergic [4,9,15-17]. Our results from *in situ* hybridization (Figure 3) shows that the mouse *Slc6a17* gene has similar expression pattern as previously seen in rat, with high expression in mouse forebrain and midbrain and lower expression in some parts of the hindbrain such as the basal ganglia. Low mRNA expression was seen in spinal cord. We confirmed *Slc6a17*gene expression in GABAergic neurons in hippocampus, in the pyramidal and granule cell layer of the dentate gyrus [41], and in the Purkinje cell layer of cerebellum [42]. The mRNA expression was also found in all layers of cerebral cortex, except layer 1, with strongest expression in cortical layer 5, results that support expression by glutamatergic neurons [43]. The layer-like pattern in cortex gives strength to the conclusion that the expression is neuronal, while a scattered pattern would suggest expression in astrocytes. Hybridization in sections of mouse spinal cord showed expression in subsets of somatic motor neurons and in interneurons. Cortical co-localization of *Slc6a17* mRNA with glutamatergic (PAG) and GABAergic (Gad67) mRNA markers confirmed *Slc6a17* staining in both excitatory and inhibitory neurons in brain (Figure 2), while co-immuno labeling with the astrocyte protein marker GFAP showed no overlap in spinal cord, suggesting no expression of *Slc6a17* in glial cells. The expression in Piris is highly interesting as this area have been shown to be part of a possible amino acid chemo-sensing system, involved in recognition of diets with deficiency of essential amino acids, producing an anorectic response [44,45]. We also found expression of *Slc6a17* in many regions involved in homeostatic control, with pronounced hypothalamic expression specifically in VMH, DMH, LH and Arc. In addition, we found expression in the amygdala (BLA), the pons (LC), and the ventral striatum (NAcc). These are all areas known to be part of the reward network and in regulation of food intake [46-49]. The *Slc6a17* mRNA expression pattern in mouse CNS strongly suggests that *Slc6a17* play a role in both excitatory and inhibitory neurotransmission and that the transporter could be involved in regulating food intake.

Here, we used a custom-made mouse B^0^AT3 antibody for further cellular localization of the B^0^AT3 protein in mouse brain and spinal cord. Immunohistological double labelling on primary cell cultures with the neuronal cell marker (MAP2) and the neuronal terminal marker (synaptophysin) visualized B^0^AT3 localization to neurons, with expression both in soma and in synapses (Figure 6). In addition, PLA studies showed protein proximity between antibodies targeting B^0^AT3 and synaptophysin, a marker for synaptic vesicles, in a hypothalamic mouse cell line (Figure 5). Localization to synapses was also confirmed with double labelling on mouse brain and spinal cord sections (Figures 4 and 5). GABAergic markers (GAD67 and VIAAT) were used and showed neuronal B^0^AT3 localization in GABAergic neurons and in GABAergic synapses. Interestingly, glutamatergic markers (PAG, VGLUT1 and VGLUT2) showed that B^0^AT3 is also localized at glutamatergic neurons and in glutamatergic synapses. Direct protein interactions between B^0^AT3 and PAG in glutamatergic neurons were shown with PLA. We further investigated whether B^0^AT3 was expressed in choroid plexus, by co-staining with pan-cytokeratin to visualizethe cellular layer surrounding the ventricles of the brain. Highly overlapping expression has previously been shown for B^0^AT2 and the choroid plexus marker (unpublished data), here B^0^AT3 and pan-cytokeratin did not co-localize. Despite the absence of co-localization within cells, B^0^AT3 still localized to the choroid plexus, but only to the brain side of choroid plexus cells. Possibly, B^0^AT3 and B^0^AT2 function within these cells and contribute to the uptake of circulating amino acids from the blood stream into the brain. B^0^AT3 has been suggested to be H^+^-dependent [11], but another study has shown that it is Na^+^-dependent [12]. Our results, showing very clear expression in synapses (most likely located in vesicles), support that B^0^AT3 is H^+^-driven, a feature typical for other vesicular transporters such as VGLUTs, VIAAT and VMAT[38,50]. However, some IHC staining presented here suggest that B^0^AT3 is also found in the soma of neurons, most likely with expression in the plasma membrane. If this expression is functionally relevant, B^0^AT3 is most likely utilizing the Na^+^-gradient over the plasma membrane to drive transport. This is supported by previous studies that have shown that, transport through B^0^AT3 can be driven by H^+^ as well as Na^+^ gradients [12].

Both acute and chronic food restriction (Figure 7) induce activation of synaptic neurotransmission in hypothalamus, which in turn boasts a strong signal to start food seeking and consumption. Starvation increases the levels of circulating ghrelin which activates neurons in hypothalamus to release the excitatory neurotransmitter glutamate [51,52]. Glutamate can in turn activate the N-methyl-D-aspartate (NMDA) receptor [53,54] located on GABAergic neurons, also expressing NPY and AgRP, and hence stimulate feeding [26]. Our results show that *Slc6a17*mRNA is highly regulated in response to starvation, suggesting that up-regulation of the transporter could be part of the enhancement of the excitatory signal during starvation (Figure 7A), while the expression of *Slc6a15* appeared to be less affected. The effect was only seen for long term food restriction, and was not observed after acute food deprivation. Up-regulation of the *Slc6a17* transporter indicates increasing uptake of amino acids into vesicles, a function that could influence a number of other mechanisms. B^0^AT3 has a broad transport profile displaying, among others, uptake of glycine. Of all transporters of amino acids, there are a number of known transporters, with high capacity for glycine transport (GlyT1 (SLC6A9), GlyT2 (SLC6A5), SNAT2 (SLC38A2), PAT1 (SLC36A1), VIAAT (SLC32A1) [3,50,55,56]), although only B^0^AT3 has vesicular expression. Here, we also study the mRNA expression of *Slc6a17* in POMC and NPY positive cells. The *Slc6a17* transporter showed localization both at hypothalamic POMC and NPY neurons (Figure 2), which is in agreement with the possible role in body weight homeostasis.

When we challenged the 5-HT (fluoxetine) and the DA/NA systems (bupropion) in rats over a treatment period of fourteen days, we found significant changes in the levels of transcripts for *Slc6a17* and *Slc6a15* (Figure 7C-D). Administration of fluoxetine, an antidepressant of the selective 5-HT reuptake inhibitor (SSRIs) type, resulted acutely in an increase of 5-HT in the synaptic cleft. Both *Slc6a17* and *Slc6a15* were significantly up-regulated in hippocampus after fluoxetine injections. Similar stimulatory effect on hippocampal and cortex expression is observed for the vesicular glutamate transporter VGLUT1 in response to antidepressant treatment with fluoxetine, and the transporter is linked to increased 5-HT levels [57,58]. Moreover, mice with reduced VGLUT1 expression show increased anxiety, depressive-like behaviors, and impaired recognition memory [59]. Bupropion acts as a dual NA and DA uptake inhibitor in several mesocorticolimbic areas [60]. The drug inhibit the reuptake of NA and DA through blockage of the NA transporter (NET/SLC6A1) and DA transporter (DAT/SLC6A3) in humans, without affecting release or transport of other neurotransmitters and without binding to other neurotransmitter receptors [61]. We showed that increased levels of DA and NA gave significant up-regulation of both *Slc6a17* and *Slc6a15* in prefrontal cortex, up-regulation of *Slc6a17* in hypothalamus and of *Slc6a15* in hippocampus. The results once again demonstrate that the expression levels of *Slc6a17* and *Slc6a15* are regulated by changes in monoamine levels. We suggest that the increase of these transporters may have a role in regulating the availability of amino acids used for neurotransmitter precursors. The *Slc6a15* results are supported by results from a previous study by Kohli *et al.*[24] where SLC6A15 was associated with major depression. Here it was found, in a whole genome association study, that allelic variants of SLC6A15 increased the risk of acquiring major depression, although the mechanism behind this is not known. Antidepressant drugs are thought to acutely increase the levels of 5-HT and NA/DA in the brain but, in long term antidepressant drugs, it has been shown to induce remodeling of neuronal circuits by strengthening of synapses [25], involving both glutamatergic and GABAergic neurons and possibly SLC6A15 and SLC6A17 could play a role in these long term effects. It is also known that starvation induces glutamatergic signaling in POMC neurons of the hypothalamus and it is possible that B^0^AT3 enhances the signaling capabilities of glutamatergic neurons. B^0^AT3 is unique among the SLC6 family of proteins in that sense that it is expressed on vesicles rather than on the plasma membrane. B^0^AT3 has been shown to transport, among other amino acids, glycine. Glycine has at least two important functions in glutamatergic neurotransmission; first, glycine can be used for the synthesis of the excitatory neurotransmitter glutamate, and secondly, glycine is a necessary co-factor for the ability of glutamate to activate NMDA receptors [53,54,62]. The up-regulation of the *Slc6a17* transporter after long term food restriction and activating drugs suggests the transporter to be involved in the increased glutamatergic signaling. It is possible that B^0^AT3 provides a mechanism to enhance NMDA receptor activation, possibly providing stronger LTP response, by packing glycine into glutamate containing vesicles and hence increase the local concentration of glycine in the synaptic cleft.

## Conclusions

In conclusion we provide an extensive localization study of B^0^AT3 (*Slc6a17*) in mouse nervous system. We show that B^0^AT3 is expressed in both excitatory and inhibitory neurons, with high expression in synapses, although we also see expression in the somato-dendritic part of neurons. We show that B^0^AT3 expression is highly overlapping with VIAAT and VGLUT2 expression, and to a lesser extent with VGLUT1. We also demonstrate that *Slc6a17*mRNA is highly regulated by administration of drugs targeting the 5-HT system and the dopamine and noradrenaline systems as well as during periods of starvation.

## Methods

### Ethical statement

All animal procedures were approved by the local ethical committee in Uppsala and followed the guidelines of European Communities Council Directive (86/609/EEC).

### RT-PCR on a panel of rat tissues

#### Animal handling and tissue isolation

Four male and two female adult Sprague–Dawley rats (Alab, Sweden) were kept under controlled environmental conditions (12 hour dark/light cycles, in an air humidity of 55% at 22°C). The rats had *ad libitum* access to water and R36 food pellets (Lactamin, Sweden). These conditions were maintained for 7 days, and at the end of the period the animals were sacrificed by decapitation between 3 and 6 h into the light period. Various brain regions and peripheral tissues (Figure 1) were isolated by dissection and two brains were dissected into cross sections, termed I-VIII in Figure 1, using a brain matrix as described in Lagerström *et al.*[63]. In short, the olfactory bulb was removed in one piece before putting the brain in the matrix (I). Slices (II-VII) were cut with razor blades (approximately 3 mm thick), with an exception for the last slice (VIII) which was slightly thinner. The tissues were immersed into RNA-later solution (Ambion, USA), kept at room temperature for 1 hour and thereafter stored at −80°C until further processed.

#### RNA preparation and cDNA synthesis

Tissue samples were homogenized by ultra-sonication in TRIzol reagent (Invitrogen, Sweden) using a sonicator (Branson Ultrasonics Corporation, USA). Chloroform was added to the homogenate, and samples were centrifuged at 12000 g for 15 min at 4°C. The aqueous phase was transferred to a new tube and isopropanol was added for RNA precipitation. Samples were incubated at −20°C over night, centrifuged at 12000 g for 10 min at 4°C to form pellets, which were washed with 75% ethanol, air dried and dissolved in RNAse-free water. DNA contamination was removed by RNase-free deoxyribonuclease 1 (Roche Diagnostics, Sweden) treatment for 3 hours at 37°C. The RNA was confirmed to be free of genomic DNA by PCR with rGapdh primers, for primer information see Table 1. Total RNA concentration was determined using a NanoDrop ND-1000 spectrophotometer (NanoDrop Technologies, USA). For cDNA synthesis, M-MLV reverse transcriptase (General Electric, Sweden) was used with random hexamers as primers according to manufacturer’s instructions. The cDNA synthesis quality was then confirmed by PCR, as described above.

**Table 1:** Primer information

**Transcript**	**Accession no.**	**Forward primer**	**Reverse primer**	**Annealing Temp.**
*H3f3b*	NM_053985	attcgcaagctcccctttcag	tggaagcgcaggtctgttttg	51°C
*Rpl19*	NM_031103	tcgccaatgccaactctcgtc	agcccgggaatggacagtcac	54°C
*Actb*	NM_031144	cactgccgcactctcttcct	aaccgctcattgccgatagtg	53°C
*Cyclo*	M19533	gagcgttttgggtccaggaat	aatgcccgcaagtcaaagaaa	51°C
*Gapdh*	X02231	acatgccgcctggagaaacct	gcccaggatgccctttagtgg	55°C
*Sdha*	NM_130428	gggagtgccgtggtgtcattg	ttcgcccatagccccagtag	53°C
*Tubb5*	NM173102	cggaaggaggcggagagc	agggtgcccatgccagagc	57°C
*Slc6a17*	NM_001033079	cagttacaacaaggacaacaac	ctgaccagaagggagatgc	53°C
*Slc6a15*	NM_172321	tgcatggatcaaggagaaggc	gcgacgaatgaaaacgactgg	58°C

#### Quantitative real-time PCR

The cDNA was analyzed with quantitative real-time PCR on MyiQ (Bio-Rad Laboratories, USA). All primers were designed with Beacon Designer v4.0 (Premier Biosoft, USA). Primers used for housekeeping genes were *H3f3b* and *Rpl19* and *Slc6a17* was the gene of interest (for primer information, see Table 1). Each real-time PCR reaction had a total volume of 20 μl and contained cDNA synthesized from 25 ng of total RNA; 0.25 μmol/ l of each primer, 20 μmol/l Tris/HCl (pH 8.4), 50 μmol/l KCl, 4 μmol/l MgCl_2_, 0.2 μmol/l dNTP, SYBR Green (1:50000 dilution). Real-time PCR was performed with 0.02 u/μl Taq DNA polymerase (Invitrogen, USA) under the following conditions: initial denaturation for 3 min at 95°C, followed by 50 cycles of 15 sec at 95°C, 15 sec at 51–54°C (optimal annealing temperature) and 30 sec at 72°C. This was followed by 84 cycles of 10 sec at 55°C (increased by 0.5°C per cycle). All real-time PCR experiments were performed in duplicates and measurements with Δ threshold C_t_-values larger than 0.99 were repeated. A negative control (water) for each primer pair and a positive control with 25 ng of rat genomic DNA was included on each plate.

#### Data analysis and relative expression calculations

The MyIQ v1.04 software (Bio-Rad Laboratories, Sweden) was used to analyze the real-time PCR melt curve data and threshold cycle C_t_-values. Melting curves were compared to the positive (genomic DNA) and negative (water) control to confirm that only one product with the expected melting point was amplified and that the product was different from the negative control. The duplicates for the raw C_t_-values were compared and excluded if the differences was greater than 0.99. The sample C_t_-values were considered expressed and further analyzed if the difference between the sample and the negative control was greater than 2. LinRegPCR v7.5 [64] was used to calculate PCR efficiencies for each sample. The average PCR efficiency and standard deviation for the primer pair was calculated after excluding outliers found with Grubbs’ test (GraphPad, USA). The delta C_t_-method was used to transform the C_t_-values into relative quantities with standard deviations. Subsequently, all values were divided by the relative quantity for genomic DNA and the GeNorm software [65] was used on the two housekeeping genes in order to calculate normalization factors for every tissue and to compensate for differences in cDNA amount. The gene of interest was normalized to the geometric mean of the expression levels of the rH3f3b housekeeping gene and the normalized quantities were then calculated to the expression relative to maximum (fold decrease) by assigning the tissue with the highest expression the value of 100% and all other levels were normalized accordingly.

### In situ hybridization and immunohistochemistry methods

#### Tissue collection and sectioning

Tissue collection and sectioning of free floating and paraffin embedded mouse brain and spinal cord sections were performed as described previously [66], with the exception that mice were anesthetized with an intra-peritoneal injection of sodium pentobarbital (90 mg/kg IP; Apoteksbolaget, Sweden) instead of Ketalar and Domitor. Cryo sections were made with fresh frozen mouse brains. Animals were sacrificed by decapitation and the brains were rapidly removed and frozen in 2-methyl-butane at −25 ± 5°C and kept at −80°C until further processing. Coronal frozen sections (14 μm) were cut using a Leica CM1800 cryostat (Leica Microsystems, Germany) at −20 ± 1°C, thaw-mounted on gelatin-coated slides and dried with a fan for 60 min before storage at −80°C.

#### Design and synthesis of RNA probes

Antisense probes were generated from commercial mouse cDNA clones (Invitrogen, USA) by using gene-specific promoters, described in Table 2. The clones were sequenced (Eurofins MWG Operon, Germany) and verified to be correct. The plasmids were linearized with restriction enzymes (Fermentas, Canada) as detailed in Table 2, and the probes were synthesized using 1 μg cleaved vector as template with T7 or T3 RNA polymerases in the presence of digoxigenin (DIG)- or fluorescein (FITC)-11-UTP (Roche Diagnostics, Switzerland). Labeled probes were quantified using the NanoDrop ND-1000 spectrophotometer (NanoDrop Technologies, USA).

**Table 2:** **Details of *in situ *hybridization probes**

**Gene symbol**	**Clone ID**	**Nucleotides**	**Accession no.**	**Restriction enz.**	**RNA pol.**
*Slc6a17*	4503453	1890-2175	NM_172271.2	BstXI	T3
*Pomc*	5024789	124-962	NM_008895.3	EcoRI	T3
*Npy*	482891	1-539	NM_023456.2	EcoRI	T3
*Glutaminase*	6838645	1638-2840	NM_001081081.2	NsiI	T3
*Gad67*	6808909	2680-3153	NM_008077.4	EcoRV	T3

#### Double in situ hybridization on cryo sections

Day 1, sections were rinsed in PBS, digested in proteinase K and fixed in 4% PFA. After washes in PBS, sections were acetylated in 1.3% trietholamine (Sigma-Aldrich, USA), 0.06% HCl (Sigma-Aldrich, USA), and 2% acetic anhydride (Fluka, Switzerland) diluted in 0.1% DEPC water. Sections were then permeabilized in 1% Triton X-100 and rinsed in PBS prior pre-hybridized in hybridization buffer (50% formamide (Sigma-Aldrich, USA), 5XSSC, pH 4.5, 5XDenhardt’s solution, 250 μg/ml of yeast transfer RNA (Sigma-Aldrich, USA), 500 μg/ml of sheared salmon sperm DNA (Ambion, USA) in 0.1% DEPC water) for 2 hours at 55°C. DIG-labeled *Slc6a17* and FITC-labeled POMC or NPY were denatured and added (0.8 μg/150 μl) for hybridization overnight at 55°C. Day 2, sections were rinsed in warm 5XSSC, incubated in 0.2XSSC for 1 hour at 55°C, washed in 0.2XSSC, and pre-blocked in blocking solution (0.1 M Tris–HCl, pH 7.5, 0.15 M NaCl, and 10% albumin bovine serum (Sigma-Aldrich, USA)) for 1 hour. Then, sections were incubated in alkaline phosphatase (AP) anti-DIG Fab fragments (1:5000, Roche Diagnostics, Switzerland) diluted in blocking solution overnight. On day 3, sections were washed in TBST with 2 mM levamisole and subsequently in NTMT with 2 mM levamisole, thereafter color was developed in Fast Red solution (Roche Diagnostics, Switzerland). Sections were treated with 5XSSC for 1 hour at 55°C and incubated in glycine-HCl for 30 min to inactivate the alkaline phosphatase. After rinsing in PBS sections were pre-blocked before incubation overnight in anti-FITC-AP Fab fragments (1:5000, Roche Diagnostics, Switzerland) diluted in blocking solution. On day 4, color development was performed with BM-Purple-AP enzyme substrate (Roche Diagnostics, Switzerland) at 37°C. Sections were mounted in 50% glycerol and analyzed using a microscope (Olympus, Japan) with an Optigrid system (Thales Optem,USA) and Volocity software (Improvision, USA).

#### Double in situ hybridization on paraffin sections

The method was performed as described for “combined *in situ* hybridization/immunohistochemistry on paraffin sections” previously described in [66], with the following exceptions: Day 1 - DIG-labeled *Slc6a17* and FITC-labeled Glutaminase or Gad67 were denatured and added (0.8 μg/150 μl) for hybridization. Day 2 - incubation in anti-FITC-POD Fab fragments (1:500, Roche Diagnostics, Switzerland). Day 3 - DAB (Sigma-Aldrich, USA) substrate was developed and sections were incubated in anti-DIG-AP Fab fragments (1:5000, Roche Diagnostics, Switzerland). Day 4 - Fast Red (Roche Diagnostics, Switzerland) was developed.

#### Combined in situ hybridization/immunohistochemistry on paraffin sections

The method was performed as described previously in [66], with some exceptions. The DIG-labeled *Slc6a17* probe was detected with BM-Purple-AP substrate enzyme, while the glial fibrillary acidic protein (GFAP) was detected with goat-anti-chicken-488 secondary antibody (for probe and antibody information see Tables 2 and 3, respectively).

**Table 3:** Details of antibodies used for fluorescent immunohistochemistry

**Primary antibodies**	**Species**	**Dilution**	**Company**
B^0^AT3	Rabbit	1:200	Custom made (Innovagen, Sweden)
B^0^AT3	Mouse	1:50	Sigma-Aldrich, USA
NeuN	Mouse	1:400	Millipore, Sweden
MAP2	Mouse	1:500	Sigma-Aldrich, USA
Gad67	Mouse	1:200	Millipore, Sweden
VIAAT	Mouse	1:300	Synaptic Systems, Germany
Synaptophysin	Mouse	1:200	BD Transduction lab, Sweden
GFAP	Chicken	1:400	AbCam, United Kingdom
Pan-cytokeratin	Mouse	1:200	Sigma-Aldrich, USA
PAG	Mouse	1:100	AbCam, United Kingdom
VGLUT1	Guinea pig	1:500	Innovagen, Sweden, based on sequences described in [40,67].
VGLUT2	Guinea pig	1:500	Innovagen, Sweden, based on sequences described in [40,67].
**Secondary antibodies**	**Species**	**Dilution**	**Company**
Anti-rabbit-594	Donkey	1:400	Invitrogen, USA
Anti-guinea pig-488	Goat	1:400	Invitrogen, USA
Anti-mouse-488	Goat	1:400	Invitrogen, USA
Anti-chicken-488	Goat	1:400	Invitrogen, USA

#### In situ hybridization on free floating sections

Single probe *in situ* hybridization was performed on free floating mouse brain and spinal cord sections as described previously [66], with some exceptions. The hybridization was performed using 1 μg/ml DIG-labeled *Slc6a17* probe diluted in hybridization buffer. Sections were mounted in 50% glycerol and photographed using a Pannoramic midi scanner and the Pannoramic viewer software v.1.14 (3DHistech, Hungary).

#### Fluorescent immunohistochemistry on paraffin embeddedsections

The method was performed as described previously in [66], with some exceptions. Sections were incubated with the custom-made polyclonal antibody rabbit-anti-B^0^AT3 (directed against the peptide sequence Ac-ELDTEDRPAWNSKLC-CONH_2_, Innovagen, Sweden) together with one of the antibody markers (NeuN, MAP2, Gad67, VIAAT, synaptophysin, GFAP, pan-cytoceratin, PAG, VGLUT1, VGLUT2) diluted in supermix (for antibody information see Table 3). Sections were photographed using a Zeiss LSM 510 Meta confocal microscope and analyzed with AxioVision Rel. 4.8 software (Zeiss, Germany) or with a fluorescent microscope (Zeiss Axioplan2 imaging) connected to a camera (AxioCamHRm) with the Carl Zeiss AxioVision v. 4.7 software.

### Primary cell cultures

#### Tissue and cell collection

Primary forebrain cell cultures were prepared from embryonic gestation day 13 (E13) mouse embryos. A pregnant female C57Bl6/J mouse was sacrificed by an overdose of CO_2_. The uterus was surgically removed from the mouse and placed in a petri dish with cold Leibovitz’s L-15 medium (Invitrogen, USA). Forebrain were isolated from embryos and transferred to a new dish containing sterile L-15 medium and kept on ice. Tissues were rinsed from meninges and cut into small segments. The suspension was pipetted up and down in a tip to further separate the tissue until a homogenous solution was obtained. Homogenous solution was applied to a cell strainer (mesh size 70 μm) for filtration to further dissociate cells and to remove large residue and debris. Dissociated cells in solution were centrifuged at 1000 rpm for 5 min. The media was removed and the pellets were resuspended in 10 ml Neurobasal medium (Invitrogen, USA). Poly-L-lysine (10 μg/ml, Sigma-Aldrich, USA) coated coverslips were added to the bottom of a 12 well plate. Cells were plated in the wells at an approximate density of 0.5 million cells/cm^2^, and incubated at 37°C with 5% CO_2_ and 92% humidity. Cell cultures were kept for 5–7 days in order for the cells to mature.

#### Immunohistochemistry on primary cultures

Cells were rinsed in PBS, fixated in 4% PFA, pre-blocked in supermix (Tris-buffered saline, 0.25% gelatin, 0.5% Triton X-100), and incubated with the primary antibodies B^0^AT3, MAP2, and synaptophysin diluted in supermix over night at 4°C (for antibody information, see Table 3). Cells were repeatedly rinsed in PBS, incubated in secondary antibodies for 2 hours, and stained with DAPI (1:2500) (Sigma-Aldrich, USA). After mounting in glycerol with anti-fade (diazabicyclo, 2.2.2) octane, images were captured on a Olympus BX61WI microscope and analyzed with Volocity software (Improvision, England), or z-stack pictures were taken on a Zeiss LSM 510 Meta confocal microscope and analyzed with AxioVision Rel. 4.8 software (Zeiss, Germany).

### Proximity ligation assay (PLA) technology

The immortalized embryonic mouse hypothalamus cell line N25/2 (mHypoE-N25/2, Cellutions Biosystems Inc., Canada) was cultured on glass slides (coated with 10 μg/ml poly-L-lycine) for 40 hours. Cells were rinsed with PBS and fixed in 4% paraformaldehyde (Sigma-Aldrich, USA) for 15 min. The Duolink II fluorescence kit (red detection reagents, Olink Biosciences, Sweden) was used to run *in situ* proximity ligation assay (PLA) on the fixed cells according to manufacturer’s instructions [68-71]. Information about primary B^0^AT3, PAG and synaptophysin antibodies is listed in Table 3. A negative control was run without primary antibodies. Protein interactions were detected with anti-rabbit PLUS and anti-mouse MINUS PLA probes. Slides were photographed using a fluorescent microscope (Zeiss Axioplan2 imaging) connected to a camera (AxioCamHRm) with the AxioVision v. 4.7 software (Carl Zeiss, Germany). PLA signals were shown as a merged image of the raw data aquired from 20 z-planes, using the Axioplane software.

### RT-PCR on tissues from food restricted rats and antidepressant drugs- treated rats

#### Animal handling and tissue isolation, food restriction

Animals used in Lindblom et al. [72] were also used in this study. Briefly, twenty-four male Sprague–Dawley rats (Alab, Sweden) with initial body weight of 223 ± 1.5 g were randomized into control, food deprived and food restricted groups (n = 8/group). The experiment was maintained for 12 days and all animals had free access to water. Food consumption was monitored daily and body weights were measured every fourth day. Rats in the control group had *ad libitum* access to R36 food pellets (Labfor, Lactamin, Sweden), whereas, the food restricted group received 45 ± 1% of the amount consumed by the control animals. Food deprived animals had *ad libitum* access to food until the last 48 hours of the experiment, when they were completely deprived of food. The experiment was maintained for 12 days and during this period the control animals gained 40 ± 1.5% in body weight, whereas the food restricted animals lost 8 ± 0.9%. The deprived rats gained in total 24 ± 0.8% in body weight. Animals were killed by decapitation, the brains were rapidly removed and tissues of interest were isolated by micro-dissection from slices obtained with a brain matrix. Samples were rapidly frozen on dry ice, immersed in RNA-later solution (Ambion, USA), kept at room temperature for 1 hour and then stored at −80°C until further processed.

#### Animal handling and tissue isolation, drug treatment

Thirty-two male Wistar rats were kept under controlled environment with *ad libitum* access to R36 food pellets and water. Rats were randomized into a saline control group, two fluoxetine groups, one low dose (3 mg/kg) and one high dose (10 mg/kg), and a high dose (25 mg/kg) bupropion group (n = 8/group). The antidepressants were dissolved in saline. Animals were subcutaneously injected with the drug once daily and the experiment were maintained for fourteen days. Animals were decapitated, brains were removed and tissues of interest were isolated by micro-dissection from slices obtained with a brain matrix. Samples were rapidly frozen on dry ice, immersed in RNA-later solution (Ambion, USA), kept at room temperature for 1 hour and then stored at −80°C until further processed.

#### RNA preparation, cDNA synthesis and real-time PCR

RNA was extracted and converted into cDNA and used as template for quantitative real-time PCR as described in “RT-PCR on panel of rat tissues”. Primers used as housekeeping gene was rCyclo, rH3f3b, rActb, rTubb5, rRpl19, rGapdh and rSdha for food restricted rats and the same set with the exclusion of rSdha for drug treated rats (for primer information, see Table 1).

#### Data analysis and relative expression calculations

The MyIQ v1.04 software (Bio-Rad Laboratories, Sweden) was used to analyze the real-time PCR melt curve data and threshold cycle C_t_-values. Melting curves were compared to the positive (genomic DNA) and negative (water) control to confirm that only one product was amplified, with the expected melting point, and that the product was different from the negative control. The duplicates for the raw C_t_-values were compared and excluded if the differences were greater than 0.99. The sample C_t_-values were considered expressed and further analyzed if the difference between the sample and the negative control was greater than 2. LinRegPCR v7.5 [64] was used to calculate PCR efficiencies for each sample. The average PCR efficiency and standard deviation for each primer pair were calculated after excluding outliers found with Grubbs’ test (GraphPad, USA). The delta C_t_-method was used to transform the C_t_-values into relative quantities with standard deviations. The GeNorm software [65] was used on the housekeeping genes in order to identify the most stable housekeeping genes and to calculate normalization factors for each tissue. The genes of interest were subsequently normalized with the normalization factors obtained from GeNorm and used for statistical analysis.

Differences in gene expression between groups were analyzed using one-way analysis of variance (ANOVA) followed by Tukey’s multiple comparison post hoc test in Prism v. 5.02 (GraphPad, USA) where appropriate p < 0.05 (95% confidence intervals) was used as criteria for statistical significance for the ANOVA.

## Competing interests

The authors declare that they have no competing interests.

## Authors’ contributions

MG, SH, SB, AJ, VN, SW, OS, SS, JR performed the experiments. MG, SH, SB, OS, SS, JR, VK, RF analysed the data. MH, SB, SS, JR, VK, RF designed the experiments. MH, RF, SB wrote the paper. All authors read and approved the final manuscript.

## Supplementary Material

Additional file 1: Figure S1 Characterization of the B0AT3 antibody. The antibody specificity was investigated by immunohistochemistry on mouse CNS tissues. Double immunohistochemistry with the custom made polyclonal B0AT3 antibody made in rabbit (red) and the commercial available B0AT3 antibody made in mouse (green) on brain (row A) and spinal cord (row B) tissue. The cell nucleus marker DAPI was stained in blue. **(A)** Co-localization of B0AT3 antibodies in cells in cerebral cortex in brain (Bregma -1.06). **(B)** Co-localization of B0AT3 antibodies in motor neurons and other cells in spinal cord (L2). Scale bar: 10 μm. The immunohistochemistry indicated that the custom made polyclonal B0AT3 antibody was epitope specific. **Figure S2.** Protein localization of B0AT3 to glutamatergic neurons and vesicles. Red staining is B0AT3, green staining is VGLUT1 and VGLUT2 respectively and blue is DAPI. **(A)** Overlapping expression between B0AT3 and VGLUT1 in cerebral cortex in the brain. **(B)** Overlapping expression for the vesicular marker VGLUT2 and B0AT3 in in cerebral cortex in the brain. **Table S1.** CNS expression of *Slc6a17* mRNA in mouse brain. The scale of estimated *Slc6a17* mRNA expression in the table; (+++) high expression, (++) medium expression, (+) low expression, and (-) not detected. Click here for file
