# Supplementary material for: Characterization of the transporterB0AT3 (Slc6a17) in the rodent central nervous system
Source: BMC Neurosci. 2013 May 14;14:54. doi: 10.1186/1471-2202-14-54 (PMC3689596; doi:10.1186/1471-2202-14-54)
Supplement: Additional file 1: Figure S1 — Characterization of the B0AT3 antibody. The antibody specificity was investigated by immunohistochemistry on mouse CNS tissues. Double immunohistochemistry with the custom made polyclonal B0AT3 antibody made in rabbit (red) and the commercial available B0AT3 antibody made in mouse (green) on brain (row A) and spinal cord (row B) tissue. The cell nucleus marker DAPI was stained in blue. (A) Co-localization of B0AT3 antibodies in cells in cerebral cortex in brain (Bregma -1.06). (B) Co-localization of B0AT3 antibodies in motor neurons and other cells in spinal cord (L2). Scale bar: 10 μm. The immunohistochemistry indicated that the custom made polyclonal B0AT3 antibody was epitope specific. Figure S2. Protein localization of B0AT3 to glutamatergic neurons and vesicles. Red staining is B0AT3, green staining is VGLUT1 and VGLUT2 respectively and blue is DAPI. (A) Overlapping expression between B0AT3 and VGLUT1 in cerebral cortex in the brain. (B) Overlapping expression for the vesicular marker VGLUT2 and B0AT3 in in cerebral cortex in the brain. Table S1. CNS expression of Slc6a17 mRNA in mouse brain. The scale of estimated Slc6a17 mRNA expression in the table; (+++) high expression, (++) medium expression, (+) low expression, and (-) not detected. [file 1471-2202-14-54-S1.pdf]

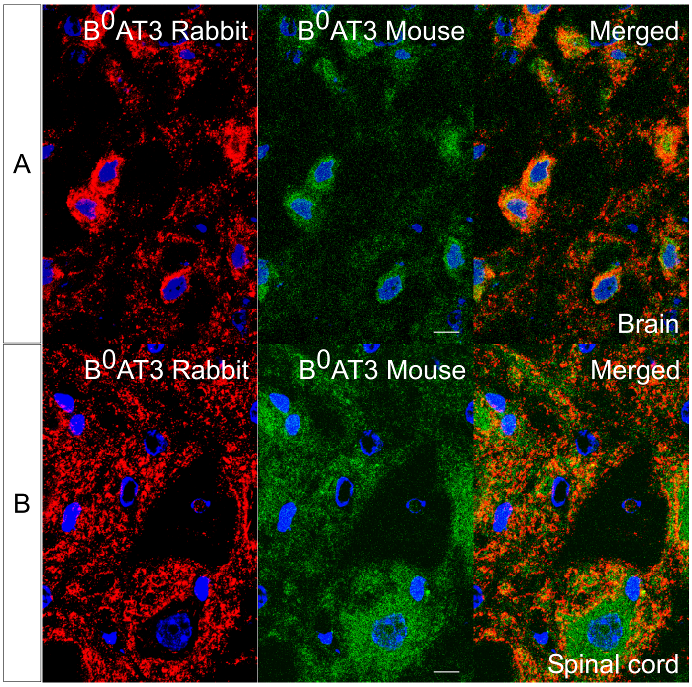

Supplementary Figure 1.

Supplementary Figure 1.

Characterization of the B^0AT3 antibody. The antibody specificity was investigated by immunohistochemistry on mouse CNS tissues. Double immunohistochemistry with the custom made polyclonal B^0AT3 antibody made in rabbit (red) and the commercial available B^0AT3 antibody made in mouse (green) on brain (row A) and spinal cord (row B) tissue. The cell nucleus marker DAPI was stained in blue. **(A)** Co-localization of B^0AT3 antibodies in cells in cerebral cortex in brain (Bregma -1.06). **(B)** Co-localization of B^0AT3 antibodies in motor neurons and other cells in spinal cord (L2). Scale bar: 10 µm. The immunohistochemistry indicated that the custom made polyclonal B^0AT3 antibody was epitope specific.

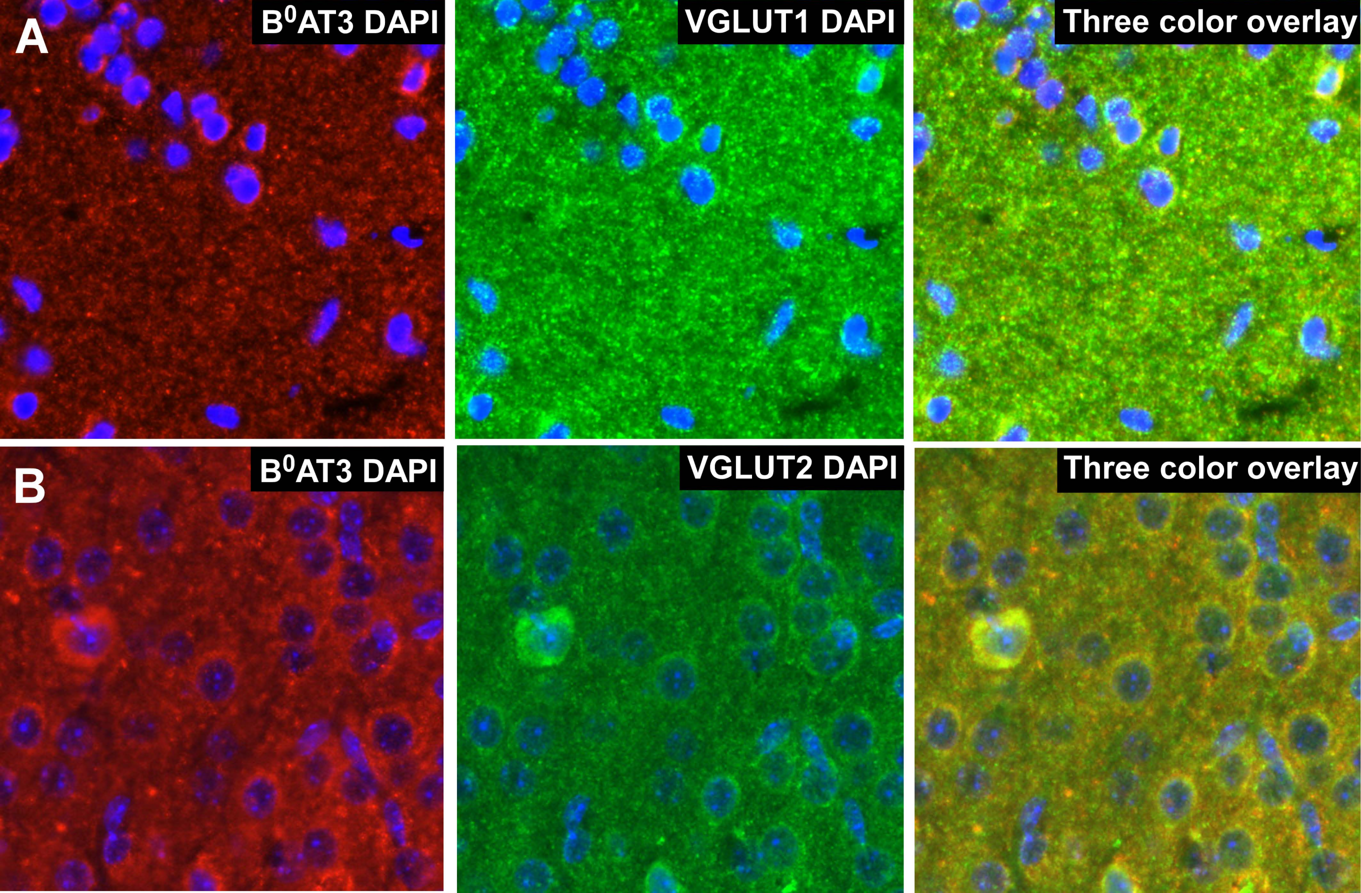
A
B⁰AT3 DAPI
VGLUT1 DAPI
Three color overlay
B
B⁰AT3 DAPI
VGLUT2 DAPI
Three color overlay

Supplementary Figure 2.

Protein localization of B^0AT3 to glutamatergic neurons and vesicles. Red staining is B^0AT3, green staining is VGLUT1 and VGLUT2 respectively and blue is DAPI. **(A)** Overlapping expression between B^0AT3 and VGLUT1 in cerebral cortex in the brain. **(B)** Overlapping expression for the vesicular marker VGLUT2 and B^0AT3 in in cerebral cortex in the brain.

Supplementary Table 1. CNS expression of *Slc6a17* mRNA in mouse brain. The scale of estimated *Slc6a17* mRNA expression in the table; (+++) high expression, (++) medium expression, (+) low expression, and (-) not detected.

**Tissue**	***Slc6a17* expression**
Stiatum	
Caudate putamen (striatum) (CPu)	+
Nucleus accumbens, core (NAccC)	+
Septum	
Lateral septal nucleus, dorsal part (LSD)	+++
Lateral septal nucleus, intermediate part (LSI)	+++
Lateral septal nucleus, ventral part (LSV)	++
Septofimbrial nucleus (SFi)	++
Ventral pallidum (VP)	++
Medial forebrain bundle (mfb)	++
Olfactory tubercle (Tu)	++
Nucleus of the vertical limb of the diagonal band (VDB)	+
Islands of Calleja (ICj)	++
Hippocampus	
Pyramidal cell layer of the hippocampus (Py)	+++
Granule cell layer of the dentate gyrus (GrDG)	+++
Cerebral cortex	
Layer 1	-
Layer 2	++
Layer 3	++
Layer 4	+
Layer 5	+++
Layer 6	+

Piriform cortex (Pir)	+++
Indusium griseum (IG)	+++
Thalamus	
Medial habenular nucleus (MHb)	++
Lateral habenular nucleus (LHb)	++
Mediodorsal thalamic nucleus (MD)	++
Laterodorsal thalamic nucleus, dorsomedial part (LDDM)	++
Laterodorsal thalamic nucleus, ventrolateral part (LDVL)	+
Central medial thalamic nucleus (CM)	+
Ventromedial thalamic nucleus (VM)	+
Ventrolateral thalamic nucleus (VL)	+
Ventral posteromedial thalamic nucleus (VPM)	+
Reticular thalamic nucleus (Rt)	+
Bed nucleus of the stria terminalis (BNST)	++
Submedius thalamic nucleus (Sub)	++
Zona incerta (ZI)	+
Hypothalamus	
Septohypothalamic nucleus (SHy)	++
Dorsomedial hypothalamic nucleus (DMH)	++
Ventromedial hypothalamic nucleus, dorsomedial part (VMHDM)	+++
Ventromedial hypothalamic nucleus, central part (VMHC)	++
Ventromedial hypothalamic nucleus, ventrolateral part (VMHVL)	++
Lateral hypothalamus (LH)	++
Arcuate hypothalamic nucleus (Arc)	+++
Paraventricular hypothalamic nucleus (Pa)	+
Anterior hypothalamic area, posterior part (AHP)	+
Amygdala	

Lateral amygdaloid nucleus, dorsolateral part (LaDL)	++
Lateral amygdaloid nucleus, ventrolateral part (LaVL)	++
Basolateral amygdaloid nucleus, anterior part (BLA)	+++
Basolateral amygdaloid nucleus, ventral part (BLV)	+++
Bed nucleus of the stria terminalis, intraamygdaloid (STIA)	+++
Basomedial amygdaloid nucleus, anterior part (BMA)	++
Dorsal endopiriform nucleus (DEn)	++
Ventral endopiriform claustrum (VEn)	++
Medial amygdaloid nucleus, posterodorsal part (MePD)	+
Medial amygdaloid nucleus, posteroventral part (MePV)	+
Central amygdaloid nucleus (Ce)	+
Pons	
Lateral parabrachial nucleus (LPB)	+
Medial parabrachial nucleus (MPB)	++
Locus coeruleus (LC)	++
Barrington’s nucleus (Bar)	++
Laterodorsal tegmental nucleus (LDTg)	+
Cerebellum	
Purkinje layer of cells	++
Spinal cord	+
